# M^3^-*S*: a genotype calling method incorporating information from samples with known genotypes

**DOI:** 10.1186/s12859-015-0824-5

**Published:** 2015-12-03

**Authors:** Gengxin Li, Hongyu Zhao

**Affiliations:** 1 Department of Mathematics and Statistics, Wright State University, 3640 Colonel Glenn Hwy, Dayton, 45435 USA; 2 Department of Biostatistics, Yale School of Public Health, 60 College Street, New Haven, 06520 USA

**Keywords:** Gaussian mixture model (GMM), Clustering, Genotype, Genotyping array, HapMap, Single nucleotide polymorphisms (SNPs), Rare SNP

## Abstract

**Background:**

A key challenge in analyzing high throughput Single Nucleotide Polymorphism (SNP) arrays is the accurate inference of genotypes for SNPs with low minor allele frequencies. A number of calling algorithms have been developed to infer genotypes for common SNPs, but they are limited in their performance in calling rare SNPs. The existing algorithms can be broadly classified into three categories, including: population-based methods, SNP-based methods, and a hybrid of the two approaches. Despite the relatively better performance of the hybrid approach, it is still challenging to analyze rare SNPs.

**Results:**

We propose to utilize information from samples with known genotypes to develop a two stage genotyping procedure, namely M^3^-*S*, for rare SNP calling. This new approach can improve genotyping accuracy through clearly defining the boundaries of genotype clusters from samples with known genotypes, and enlarge the call rate by combining the simulated data based on the inferred genotype clusters information with the study population.

**Conclusions:**

Applications to real data demonstrates that this new approach M^3^-*S* outperforms existing methods in calling rare SNPs.

**Electronic supplementary material:**

The online version of this article (doi:10.1186/s12859-015-0824-5) contains supplementary material, which is available to authorized users.

## Background

Genome-wide association studies (GWAS) have successfully identified tens of thousands of genetic variants contributing to hundreds of human diseases in the past decade [1, 2]. Their success is largely due to the availability of affordable and reliable SNP arrays, such as those from Affymetrix and Illumina [3, 4]. Computationally efficient and accurate genotyping algorithms are needed to infer genotypes of SNPs from the observed data produced by two platforms. Many calling algorithms have been developed for these two platforms, such as: Iluminus [5], GenoSNP [6], GenCall [7], CRLMM [8, 9], BEAGLECALL [10] and zCall [11] for Illumina arrays; and RLMM [12], BRLMM [13] and CHIAMO [14, 15] for Affymetrix GeneChips.

In this article, we focus on the analysis of Illumina arrays, which use two color single base extension (SBE) biochemistry technology [16] to infer the genotype of a SNP with two alleles *A* and *B*. The goal of genotype calling is to infer the genotype (AA, AB, or BB) carried by an individual across the SNPs in the genome. All the genotype calling algorithms share the common feature of first defining genotype clusters and then assigning individuals to these clusters. They differ in how the clusters are defined and how the samples are allocated to these clusters. One class of genotyping algorithms is population-based where all individuals are genotyped SNP-by-SNP. The genotype clusters are defined through the joint analysis of all samples at a given SNP separately. Although commonly used, its performance highly depends on the size of the study population. It may not perform well for SNPs with low minor allele frequencies (MAF) where there may be very few individuals for certain clusters. For example, GenCall [7], a representative method in this class, needs a large reference population (e.g. 10,000) to accurately define genotype clusters for SNPs with MAF < 0.01 [6]. In contrast, another approach, called the SNP-based method, defines genotype clusters using all SNPs of an individual at a time, as represented by GenoSNP [6]. The good performance of this approach depends on two assumptions: (1) The probes of all the SNPs behave similarly; and (2) the variations within a genotype cluster are much smaller than that between clusters. Compared to the population-based method, GenoSNP does not need a large number of samples to ensure calling accuracy for SNPs with low MAF. However, empirical applications of this approach lead to many more SNPs failing the Hardy-Weinberg (HW) principle, indicating that the two implicit assumptions are likely violated in reality.

To address the limitation of these two approaches, we developed M^3^ that combines the population-based strategy with the SNP-based approach to improve calling accuracy for rare SNPs [17]. Compared to GenCall, M^3^ borrows genotype cluster information from reference SNPs to improve the calling performance for rare SNPs. It also improves upon GenoSNP by utilizing the population-based calling scheme. However, the effectiveness of M^3^ depends on the quality of the reference SNP. If the reference SNP behaves very different from the target rare SNP, the inferred genotype will likely be incorrect. In this article, we consider using additional information to further improve the quality of the reference SNP. In particular, we consider the use of samples with known genotypes, e.g. HapMap samples, that are often included for quality control purposes. Because the HapMap samples have been extensively studied and their true genotypes can be considered known with almost certainty, the genotype calling results from these samples can provide a good metric for the performance of calling algorithms. Hence, we incorporate known genotype information from these quality control samples into the reference SNP selection procedure under the general framework for M^3^, and this new method is named M^3^-*S* where *S* denotes samples with known genotypes for calling. More specifically, M^3^-*S* utilizes known genotype information to construct three genotype clusters in the first stage, and the entire samples together with simulated data based on the inferred cluster information are then genotyped in the second stage.

The key component in our improved method is to explicitly define the boundaries of the three genotype clusters for each SNP through samples with known genotypes. Although the idea of leveraging subjects with known genotype information is intuitive, there is a practical challenge in implementations, e.g., there is often only two or even one cluster for known genotype samples, making the inference of boundaries difficult. This can be solved by taking advantage of the reference SNP selection method [17] developed for M^3^ to the samples with known genotypes. It can directly define boundaries of genotype clusters before genotyping other study subjects. In addition, our proposed method is computationally efficient and applicable to the large-scale intensity data (see Additional file 1B).

The rest of the paper is organized as follows. We first describe the two stages of our new method (M^3^-*S*), and then explain how this new method helps calling for rare SNPs. Finally we compare the performance of our method with existing methods to demonstrate its better performance.

## Methods

### Illumina chip data description

The Illumina microarrays probe millions of SNPs per sample, with newer arrays including more recently discovered rare SNPs. In their probe design, the Illumina arrays are made up of a number of beadpools containing millions of beadtypes. Every SNP is represented by one beadtype with 20 pairs of allele-specific intensities for one individual [16], thus each beadtype is able to assay both SNP alleles. In this study, we consider the pair of raw intensity values at each beadtype for every sample to infer geneotypes.

### Statistical methods

#### Stage I: Estimation of three genotype clusters from samples with known genotypes

In the first stage, samples with known genotypes are first analyzed separately to infer three genotype clusters for each SNP denoted by AA, AB, and BB. We use B to denote the less common allele, and all possible genotypes are denoted as: AA, AB, or BB. Let *x*_*is*_=(*r*_*is*_,*g*_*is*_) denote the measured intensity values of the *s* ^*th*^ SNP for the *i* ^*th*^ individual where (*i* = 1, …, *n*; *s* = 1, …, *S*) with *S* being the total number of SNPs and *n* being the total number of subjects. For every SNP, we use *a*_*hs*_=(*r*_*hs*_,*g*_*hs*_) to denote the raw intensity values of the *h* ^*th*^ subject with known genotype for the *s* ^*th*^ SNP, where *h* = 1, …, *n*_*a*_ and *n*_*a*_ is the total number of samples with known genotypes. In the study that motivated our work, HapMap samples were included and we obtained their “true” genotypes from the International HapMap Project [3, 4]. From the notations introduced above, it is clear that *n*_*a*_ is less than *n*, and each element in **M** = {1,…,*n*_*a*_} can be matched to one unique element in a set **N** = {1,…,*n*}. To distinguish SNPs with study population and SNPs only containing individuals with known genotypes, the latter is named as featured SNPs. We propose the following five-step procedure to estimate the parameters of the genotype clusters.

- (1) Step I: randomly assign samples with known genotypes into two groups. One will be called the training set with *l*_*a*_ samples, and the other is the testing set. Generally, the training samples are used to infer the parameters of genotype clusters, and the testing individuals are used to evaluate the performance of the method. In this study, we define different allocation ratios between the training samples and testing samples (ratio 1:1 or 2:1) to evaluate the impact of the allocation ratio on genotyping. For the data set that motivated this research, there are 141 samples with known genotypes. Under the allocation ratio 1:1, we consider 71 training samples, i.e. *l*_*a*_=71, and 70 testing samples. For the allocation ratio 2:1, 94 subjects are assigned to the training group, i.e. *l*_*a*_=94, and 47 individuals are in the testing set.
- (2) Step II: evaluate the quality of each featured SNP via analyzing the training samples. When the training samples are randomly selected, the number of distinct genotypes and the sample size of each genotype can be inferred from known genotypes, then all the featured SNPs with training subjects ($\boldsymbol {a}_{s}^{\ast }$: the raw intensity vector of training samples) can be classified into two groups, where G_1_ collects those featured SNPs having three distinct genotypes, whereas G_2_ collects those featured SNPs with fewer than three distinct genotypes.
  (1) $${} \left\{ \begin{aligned} \boldsymbol{a}_{s}^{\ast} \in {G}_{2} &\quad\text{if} {c}_{as}<3 \text{or} {l}_{a_{0}s}<3 \text{or} {l}_{a_{1}s}<3 \text{or} {l}_{a_{2}s}<3\\ \boldsymbol{a}_{s}^{\ast} \in G_{1} &\quad\text{otherwise } \end{aligned}\right.  $$
  where *c*_*as*_ denotes the number of genotype clusters for training samples at the *s* ^*th*^ featured SNP; $l_{a_{0}s}$, $l_{a_{1}s}$ and $l_{a_{2}s}$ denote the number of training subjects in the three genotype groups (AA, AB or BB) for the *s* ^*th*^ featured SNP.
- (3) Step III: select reference featured SNPs for target featured SNP in *G*_2_. The featured SNPs with training samples having three clear clusters are selected as candidate reference featured SNPs denoted by Ref featured SNPs. These Ref featured SNPs are searched from the featured SNPs near around the target featured SNP, and the size of search area denoted by *R* represents the total number of candidate Ref featured SNPs.
- (4) Step IV: calculate the Mahalanobis distance between the target featured SNP with training samples and each Ref featured SNP containing the same training samples. Here, the Mahalanobis distance measures the similarity between the target featured SNP and each Ref featured SNP, where the optimal Ref featured SNP is selected based on minimizing this distance. To simplify the calculation, when the *s* ^*th*^ featured SNP is the target featured SNP, the two dimensional raw intensity vector $a_{\textit {hs}}^{\ast }=(r_{\textit {hs}}, g_{\textit {hs}})$ is projected to a univariate variable *b*_*hs*_ [5], and the *s* ^*th*^ featured SNP and all Ref featured SNPs are classified into three genotype clusters in terms of this univariate variable.
  $$\begin{aligned} & b_{hs}=\frac{r_{hs}-g_{hs}}{r_{hs}+g_{hs}} \hspace{1.2em} \mathrm{s}=1, \ldots, \mathrm{S}\\ & b_{hr}=\frac{r_{hr}-g_{hr}}{r_{hr}+g_{hr}} \hspace{1.2em} \mathrm{r}=1, \ldots, \mathit{R}\\ \end{aligned} $$
  The minimum Mahalanobis distance (*d*_*s*_) is obtained by the following equation,
  $$d_{s}=\underset{r; r\in S_{R}}{min}\left\{\sqrt{\sum_{h=1}^{l_{a}}\frac{(b_{hs}-b_{hr})^{2}}{{s_{h}^{2}}}} \right\}. $$
  Note that *S*_*R*_ is the set of Ref featured SNPs selected for the *s* ^*th*^ featured SNP; *b*_*hs*_ and *b*_*hr*_ are the projected intensities of the *h* ^*th*^ training sample for the *s* ^*th*^ featured SNP and the *r* ^*th*^ Ref featured SNP separately; and *s*_*h*_ is the standard deviation of *b*_*hs*_ and *b*_*hr*_. So, the best Ref featured SNP selected through *d*_*s*_ may be most informative for the clustering of the *s* ^*th*^ featured SNP.
- (5) Step V: estimate parameters of three genotype clusters from the training individuals. When $\boldsymbol {a}_{s}^{\ast }\in G_{1}$, the parameters of 3 clusters are directly inferred from training samples of the *s* ^*th*^ featured SNP. If $\boldsymbol {a}_{s}^{\ast } \in G_{2}$, the three genotype clusters are estimated by training samples from the *s* ^*th*^ featured SNP and the best Ref featured SNP (such as: the *r* ^*th*^ Ref featured SNP). In the second case, the training samples in the *s* ^*th*^ featured SNP are not adequate to construct the three genotype clusters, and an appropriate Ref featured SNP could help the *s* ^*th*^ featured SNP estimate three cluster information. A new combined matrix ($\mathbf {aa}_{s}^{\ast }$) is defined to collect the training samples for the *s* ^*th*^ featured SNP and the *r* ^*th*^ Ref featured SNP where $\mathbf {a}_{s}^{\ast }$ and $\mathbf {a}_{r}^{\ast }$ are the raw intensity matrices of the training samples at the *s* ^*th*^ and *r* ^*th*^ SNPs, respectively.
  $$\mathbf{aa}_{s}^{\ast}=\left(\begin{aligned} \mathbf{a}_{s}^{\ast}\\ \mathbf{a}_{r}^{\ast} \end{aligned}\right) $$
  Then the combined vector of raw intensities is partitioned into three clusters according to the training samples’ known genotypes. If *k* denotes the cluster label with values 1, 2, or 3, $\mathbf {aa}_{\textit {sk}}^{\ast }$ is the combined raw intensity matrix in the *k* ^*th*^ genotype group. The mean and variance of the three genotype clusters can be estimated by the following equations,
  (2) $$ \mu_{ask}=\left\{ \begin{aligned} & (l_{ask}+l_{ark})^{-1}1_{G_{2}}^{T} \mathbf{aa}_{sk}^{\ast} \hspace{1.2em} \text{if} \boldsymbol{a}_{s}^{\ast} \in G_{2}\\ &{l_{ask}}^{-1}1_{G_{1}}^{T} \mathbf{a}_{sk}^{\ast} \hspace{5.4em} \text{if} \boldsymbol{a}_{s}^{\ast} \in G_{1}\\ \end{aligned}\right.  $$
  where *l*_*ask*_ and *l*_*ark*_ denote the number of training samples at the *k* ^*th*^ cluster for the *s* ^*th*^ featured SNP and *r* ^*th*^ Ref featured SNP, respectively; $1_{G_{2}}$ is an (*l*_*ask*_+*l*_*ark*_) x 1 column vector with all elements equal to 1; $1_{G_{1}}$ is a *l*_*ask*_ x 1 column vector with all elements equal to 1; and $\mathbf {a}_{\textit {sk}}^{\ast }$ is the raw intensity matrix of training subjects in the *k* ^*th*^ genotype group for the *s* ^*th*^ featured SNP.
  (3) $${} \mathbf{\Sigma}_{ask}=\left\{ \begin{aligned} & \frac{(\mathbf{aa}_{sk}^{\ast}-1_{G_{2}}\mu_{ask})^{T}(\mathbf{aa}_{sk}^{\ast}-1_{G_{2}}\mu_{ask})}{l_{ask}+l_{ark}-1} \hspace{1.2em} \text{if} \mathbf{a}_{s}^{\ast} \in G_{2}\\ & \frac{(\mathbf{a}_{sk}^{\ast}-1_{G_{1}}\mu_{ask})^{T}(\mathbf{a}_{sk}^{\ast}-1_{G_{1}}\mu_{ask})}{l_{ask}-1} \hspace{2.0em} \text{if} \mathbf{a}_{s}^{\ast} \in G_{1}\\ \end{aligned}\right.  $$
  Note that *μ*_*ask*_ is an 1 × 2 vector measuring the average intensity of training samples in the *k* ^*th*^ cluster for the *s* ^*th*^ featured SNP; **Σ**_*ask*_ is a 2 × 2 covariance matrix of the *k* ^*th*^ cluster at the *s* ^*th*^ featured SNP.
  In summary, the first stage focuses on selecting reference featured SNPs to better estimate the parameters of the three genotype clusters.

#### Stage II: Gaussian mixture model for augmented intensity data

In general, enlarging sample will lead to improved genotyping results, especially for rare variants. But, this is not feasible due to constrains on available samples and budget. So we propose to “increase” the sample size through simulating from the inferred cluster parameters and combine the simulated data with the observed data to improve calling accuracy in our second stage analysis.

- (1) Step I: simulate intensity data according to the inferred parameters of the three genotype clusters from the training samples with known genotypes.
  (4) $$ \begin{aligned} &\mathbf{y}_{js} \sim {Gaussian}_{k}(\mu_{ask}, \mathbf{\Sigma}_{ask}) \hspace{.5em}\text{with probability} \frac{1}{3} \\ &{j} = 1,\ldots,m, {s} = 1,\ldots,{S}, {k} = 1, 2, \text{or} 3 \end{aligned}  $$
  In this study, we simulate *m* additional individuals at each SNP from the above Gaussian mixture distribution. Parameters *μ*_*ask*_ and **Σ**_*ask*_ (*k* = 1, 2, or 3) could adequately provide the center and variability of the three genotype clusters for each SNP, and each cluster contains equal number of simulated subjects ($\frac {m}{3}$). Here, we vary the value of *m* to be 600, 1500, and 3000 to evaluate the impact of this simulated data on genotyping. Specifically, we simulate equal numbers of subjects in every genotype group to improve the representation of rare genotypes in the samples for better genotype calling. More importantly, adding this simulated data with equal numbers in each genotype cluster to the observed data will not influence the configure of major homozygote and minor homozygote for the observed data.
- (2) Step II: genotype calling using both observed and simulated data.
  The pair of original raw intensities are *x*_*is*_=(*r*_*is*_,*g*_*is*_), and the pair of simulated raw intensities are *y*_*js*_=(*r*_*js*_,*g*_*js*_), then the combined raw intensity values at the *s* ^*th*^ SNP ${\mathbf {t}}_{s} = {{\mathbf {x}_{s}}\choose {\mathbf {y}}_{s}} $ consist of the augmented data. Within one SNP, pairs of raw intensities primarily consist of three genotype clusters which correspond to three genotypes (AA, AB, and BB). We apply the Gaussian Mixture Model (GMM) with fixed components [18], to **t**_*s*_, where the number of components is fixed at three. Besides, we introduce a null component for those individuals whose genotypes are difficult to be assigned to one of the three clusters. In principle, this model assigns the *w* ^*th*^ pair of the combined raw intensities *t*_*ws*_ to one component with probability *π*_*sk*_ where *k* measures three components corresponding to the three genotypes. The latent genotype class is denoted by the indicator variable *z*_*ws*_ generated from a multinomial distribution where *z*_*ws*_ takes the value of 1, 2, or 3. Then the three-component GMM can be expressed as:
  (5) $$ \begin{aligned} &\mathbf{z}_{ws} \sim {Mult}_{3}(1,\mathbf{\pi}_{s1},\mathbf{\pi}_{s2},\mathbf{\pi}_{s3})\\ &\ell(\mathbf{t}_{s}|\boldsymbol{\Theta}_{s},\mathbf{z}_{s})=\prod_{w=1}^{n^{\ast}}\prod_{k=1}^{3}\Phi({t}_{ws}|\mathbf{\mu}_{sk},\mathbf{\Sigma}_{sk})^{I(\mathbf{z}_{ws}=k)}\\ &{\textit{w} = 1,\ldots,n^{\ast}, \textit{s} = 1,\ldots,\textit{S}, \textit{k} = 1, 2 \text{or} 3} \end{aligned}  $$
  where *n* ^∗^ is the total number of individuals collected at the *s* ^*th*^ SNP and simulated data where *n* ^∗^=*n*+*m*, and *S* is the total number of SNPs. The normal density *Φ* has mean **μ**_*sk*_ and variance-covariance matrix **Σ**_*sk*_ in the *k* ^*th*^ cluster for the *s* ^*th*^ augmented SNP data; all pairs of raw intensity at the *s* ^*th*^ augmented data are measured by **t**_*s*_= (*t*_1*s*_, $t_{2s},\ldots, t_{n^{\ast }s})^{T}\phantom {\dot {i}\!}$; the unknown parameters of the GMM is denoted by ***Θ***_*s*_= (***π***_*s*_,***μ***_*s*_,***Σ***_*s*_) where ***π***_*s*_= (*π*_*s*1_, *π*_*s*2_, *π*_*s*3_), ***μ***_*s*_=(**μ**_*s*1_,**μ**_*s*2_,**μ**_*s*3_), and ***Σ***_*s*_=(**Σ**_*s*1_,**Σ**_*s*2_,**Σ**_*s*3_).
  Through solving the score equation, the maximum likelihood estimates (MLEs) of the above parameters can be easily estimated [18]. The (*u*+1)^*th*^ iteration of the indicator variable *z*_*ws*_=*k* (k=1,2,or 3) is inferred by
  (6) $$ f_{k}({t}_{ws};\boldsymbol{\Theta}_{s}^{u})=\frac{\pi_{sk}^{u}\Phi({t}_{ws};\mathbf{\mu}_{sk}^{u},\mathbf{\Sigma}_{sk}^{u})}{\sum_{o=1}^{3}\pi_{so}^{u}\Phi({t}_{ws};\mathbf{\mu}_{so}^{u},\mathbf{\Sigma}_{so}^{u})}.  $$
  The relevant iterative estimates for the mean **μ**_*sk*_ and variance-covariance matrix **Σ**_*sk*_ are
  (7) $$ \mathbf{\mu}_{sk}^{u+1}=\frac{\sum_{w=1}^{n^{\ast}} f_{k}({t}_{ws};\boldsymbol{\Theta}_{s}^{u}){t}_{ws}}{\sum_{w=1}^{n^{\ast}}f_{k}({t}_{ws};\boldsymbol{\Theta}_{s}^{u})}.  $$
  (8) $$ \mathbf{\Sigma}_{sk}^{u+1}=\frac{\sum_{w=1}^{n^{\ast}} f_{k}({t}_{ws};\boldsymbol{\Theta}_{s}^{u})(t_{ws}-\mathbf{\mu}_{sk}^{u+1})({t}_{ws}-\mathbf{\mu}_{sk}^{u+1})^{T}}{\sum_{w=1}^{n^{\ast}}f_{k}({t}_{ws};\boldsymbol{\Theta}_{s}^{u})}.  $$
  Two values, Posterior Rate (PR: $q_{\textit {ws}}^{k}$) and Average Posterior Rate (APR: *q*_*s*_), for the *s* ^*th*^ augmented data are calculated to quantify the quality of SNP calling [17], where
  (9) $$ q_{ws}^{k}=\frac{P({t}_{ws}|k)\pi_{sk}}{\sum_{o=1}^{3}P({t}_{ws}|o)\pi_{so}}, \hspace{1em} q_{s}=\frac{\sum_{k=1}^{3}\sum_{w=1}^{n_{k}^{\ast}}q_{ws}^{k}}{\sum_{k=1}^{3}n_{k}^{\ast}}  $$
  It can be seen that PR, measures the strength of each observation’s cluster signal, and APR is the average value of all individuals’ PRs at the *s* ^*th*^ augmented data [17]. Note that $n_{k}^{\ast }$ is the sample size in the *k* ^*th*^ cluster for the *s* ^*th*^ augmented SNP data. Genotypes can be inferred from the augmented data, including both observed and simulated subjects.

## Results and discussion

### Data set description and cutoffs setting

We analyzed Illumina Omni 1M array data collected from 3258 samples to compare the performances of M^3^-*S* with representative calling algorithms, including GenCall (a population-based method), GenoSNP (a SNP-based approach), and *M* ^3^ (a hybrid of the previous two approaches). In this data set, 38 HapMap samples were measured multiple times, using a total of 141 arrays. We focused on SNPs from chromosome 22 with a total of 15,020 SNPs. The performance of each genotyping method was evaluated by the comparison results between SNP calls inferred by each calling method and those from the International HapMap Project for these HapMap samples [3]. We chose the following cutoffs for the four calling algorithms: GC score ≥ 0.15 in GenCall used to filter good quality SNPs, samples with posterior probability > 85 % for GenoSNP, and average posterior probability > 0.85 for both *M* ^3^ and *M* ^3^-*S*. The effects of different thresholds on genotyping are summarized in Additional file 1E.

### Data analysis results

Because there were 141 HapMap subjects, we used their “true” genotypes to evaluate the accuracy of different calling algorithms. We varied the numbers of training and testing samples (allocation design: 2:1 and 1:1) to explore their impacts on genotyping. The effectiveness of two allocation designs is summarized in Table 1. It is clear that the allocation 2:1 design provides more accurate genotypes compared to those of the allocation design 1:1. It is partially due to the fact that more samples are assigned to the training set to infer the boundaries of three genotype clusters under 2:1 design. Therefore, we select 2:1 design to do further analysis. Table 1 provides the comparison results among different calling methods in terms of calling accuracy. It can be seen that *M* ^3^-*S* (99.38 %) has the best call accuracy and high call rate, followed by M^3^, GenoSNP and GenCall. We can also see that M^3^ gives the highest call rate (99.77 %), followed by M^3^-*S*, GenoSNP, and GenCall.

**Table 1:** Comparisons of call rates and concordance on HapMap samples for two allocation designs

Design	Item	GenCall	GenoSNP	M^3^	M^3^-*S*
1:1	Call Rate	96.60	99.13	99.76	99.64
	Accuracy	96.41	98.47	99.23	99.33
2:1	Call Rate	96.57	99.15	99.77	99.65
	Accuracy	96.39	98.49	99.24	99.38

Note: 2:1: 94 individuals are in the training set, and 47 subjects are in the testing group; 1:1: 71 individuals are in the training set, and 70 subjects are in the testing group; M^3^-*S*: M^3^ incorporating samples with known genotypes; Call Rate: the percentage of valid genotypes; Accuracy: the percentage of consistent genotype

We simulated different sizes of samples (e.g. 600, 1500, or 3000) in the second stage of M^3^-*S* to see the impact of simulated data on the genotyping, especially for rare variants. 600, 1500, or 3000 simulated samples are roughly 1, 1/2 or 1/5 times the original study population. In brief, the performance of our proposed calling algorithm based on three different simulation designs is evaluated through the comparison between the genotypes of testing HapMap samples inferred from M^3^-*S* and the genotypes of these subjects inferred from the HapMap project. Table 2 shows that enlarging the number of samples for simulated data can improve the accuracy of genotypes of testing HapMap samples for extremely rare SNPs. Thus, the 2:1 allocation design with 3000 simulated samples gives the best genotype accuracy (99.23 %), and the highest call rate (99.81 %) for extremely rare SNPs (MAF < 0.01). For the real data, we think the 2:1 allocation design with 3000 simulated samples is preferred while using M^3^-*S* to infer genotypes. Because GenoSNP, *M* ^3^ and *M* ^3^-*S* have been developed to focus on calling less common SNPs, Table 3 summarizes the comparison results among four methods applied to these testing HapMap subjects in terms of different MAF cut-offs for SNPs. Overall, M^3^-*S* has the best call accuracy (99.22 *%* *~* 99.28 *%*) and high call rate (99.59 *%* *~* 99.81*%*) for rare SNPs with MAF < 0.1, 0.05, and 0.01, followed by M^3^, GenoSNP and GenCall.

**Table 2:** Comparisons of call rates and concordance on HapMap samples for rare variants under 600, 1500, and 3000 simulated observations and the allocation design 2:1

SNPs	# SNP	Item	M^3^-*S*	M^3^-*S*	M^3^-*S*		
			600	1500	3000		
MAF < 0.1	4364	Call Rate	99.69	99.65	99.59		
		Accuracy	99.33	99.24	99.22		
MAF < 0.05	2329	Call Rate	99.72	99.71	99.68		
		Accuracy	99.34	99.26	99.28		
MAF < 0.01	597	Call Rate	99.59	99.86	99.81		
		Accuracy	99.04	99.06	99.23		

Note: M^3^-*S*: M^3^ incorporating samples with known genotypes; Call Rate: the percentage of valid genotypes; Accuracy: the percentage of consistent genotype; # SNP: the number of SNPs whose MAFs are less than 0.1, 0.05 or 0.01, respectively

**Table 3:** Comparisons of call rates and concordance on HapMap samples for rare variants among GenCall, GenoSNP, M^3^ and M^3^-*S*

SNPs	# SNP	Item	GenCall	GenoSNP	M^3^	M^3^-*S*
MAF < 0.1	4364	Call Rate	95.89	99.02	99.70	99.59
		Accuracy	95.65	98.28	99.19	99.22
MAF < 0.05	2329	Call Rate	96.44	98.89	99.64	99.68
		Accuracy	96.15	98.02	99.08	99.28
MAF < 0.01	597	Call Rate	94.37	98.90	99.53	99.81
		Accuracy	93.89	97.28	98.60	99.23

Note: M^3^-*S*: M^3^ incorporating samples with known genotypes; Call Rate: the percentage of valid genotypes; Accuracy: the percentage of consistent genotype; # SNP: the number of SNPs whose MAFs are less than 0.1, 0.05 or 0.01, respectively

**Fig. 1:**
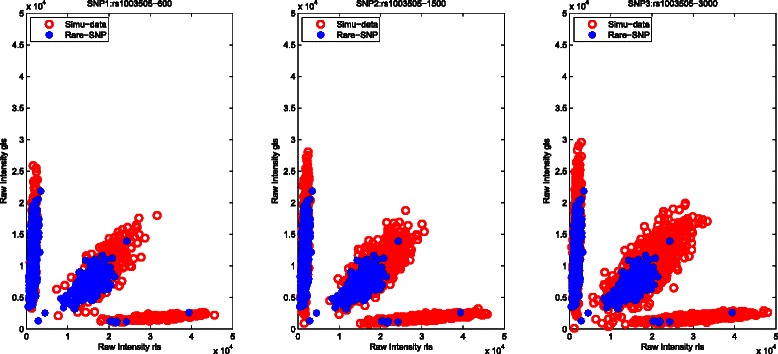
Illustration of how different sizes of simulated data improve the calling results of one rare SNPs (rs1003505)

**Fig. 2:**
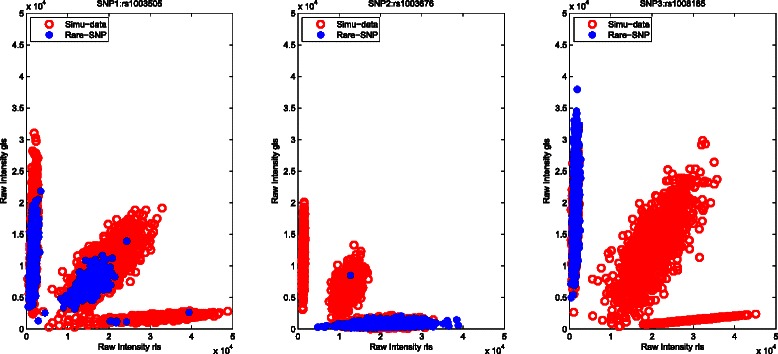
Illustration of how M^3^-*S* improves the calling results of three rare SNPs (rs1003505, rs1003676, and rs1008185)

The successful application of this proposed calling procedure (M^3^-*S*) depends on the accurate estimations of the three genotype clusters from the subjects with known genotypes in the first stage, and adequately simulated subjects from the Gaussian mixture distribution in the second stage. For parameter estimations of the three genotype clusters, the reference SNP selection method [17] may help infer the boundaries of all genotype clusters for rare SNPs. To evaluate the influence of the simulated data, we test the performances of different sizes of simulated data (e.g. 600, 1500 or 3000) on the same rare SNP (rs1003505). As shown in Fig. 1, a larger size of simulated data generates a bigger cluster easily covering the target rare SNP. Hence, adding 3000 simulated data in our real data is a good option for improving genotyping accuracies. Next, we also select three rare SNPs (rs1003505, rs1003676 and rs1008185) displaying three genotype clusters, two clusters, and one cluster, respectively. As shown in Fig. 2, our method with 3000 simulated observations can accurately infer genotypes for different rare SNPs by leveraging information from the simulated data.

Here, we extend our analysis to the entire study population, not just testing HapMap samples. For all study subjects, the call rate and the concordance rate of the four calling algorithms are compared, where the call rate is the ratio of genotypes that can be inferred from each calling method to those that need to be genotyped, and the concordance rate is the genotype agreement between two algorithms. The overall comparison results are summarized in Table 4. It can be seen that genotypes inferred from M^3^-*S*, M^3^, GenCall and GenoSNP are highly consistent, especially those from M^3^-*S* and M^3^. This is likely due to the fact that both M^3^-*S* and M^3^ are population-based calling approaches in a broad sense, and the reference SNP selection step in M^3^-*S* and M^3^ can improve their call rates (99.56 %, 99.71 %) (Table 4). Besides, M^3^ has the highest call rate because it utilizes two SNPs’ subjects (2 × 3258) to infer genotypes at rare SNPs, but M^3^-*S* uses one SNP’s individuals plus 3000 simulated subjects (3258+3000) to infer genotypes at these rare SNPs. The change in sample size of two methods results in the difference of call rate between these two approaches. Moreover, Additional file 1: Table S4 and Figure S1 further explain the differences among M^3^-*S*, GenCall and GenoSNP.

**Table 4:** Comparisons of call rate and concordance of whole SNPs among GenCall, GenoSNP, M^3^ and M^3^-*S*

Algorithm 1	Algorithm 2	Call rate (%)		Concordance (%)
		Algorithm 1	Algorithm 2	
GenCall	GenoSNP	96.71	99.12	99.71
GenCall	M^3^	96.71	99.71	99.85
GenCall	M^3^-*S*	96.71	99.56	99.85
GenoSNP	M^3^	99.12	99.71	99.41
GenoSNP	M^3^-*S*	99.12	99.56	99.45
M^3^	M^3^-*S*	99.71	99.56	99.57

Note: The unit of Call Rate and Concordance Rate is percentage %; M^3^-*S*: M^3^ incorporating samples with known genotypes; Algorithm: four algorithms in this table, that is, GenCall, GenoSNP, M^3^ and M^3^-*S*

Hardy-Weinberg Equilibrium (HWE) test is an important criterion to examine genotyping quality as failing HWE test may indicate calling errors. We performed HWE tests for the four population groups: Hispanic African-American, non-Hispanic African-American, Hispanic European-American, and non-Hispanic European-American, separately. Table 5 summarizes the number of SNPs that fail the HWE test. GenoSNP has the largest number of SNPs failing HWE test, whereas GenCall has the smallest number of SNPs failing HWE. M^3^ and M^3^-*S* fall in between in terms of the number of SNPs not meeting HWE. Specifically, we find that M^3^-*S* may make more SNPs fail the HWE test when this approach enlarges the number of samples for simulated data. It seems that improving call accuracy for rare SNPs is in conflict with guaranteeing the quality of SNPs via HWE test. People have to select the appropriate samples size for simulated data to balance the above two criteria.

**Table 5:** Comparisons of Hardy-Weinberg Equilibrium Test among GenCall, GenoSNP, M^3^ and M^3^-*S*

Population	Num-Sample	GenCall	GenoSNP	M^3^	M^3^-*S*	M^3^-*S*	M^3^-*S*
					600	1500	3000
AA I	2005	224	907	432	481	646	822
AA II	83	20	255	64	59	61	65
EA I	867	486	1024	636	639	690	770
EA II	158	40	348	109	98	106	129

Note: AA I: African-Americans not of Hispanic Origin; AA II: African-Americans of Hispanic Origin; EA I: European Americans not of Hispanic Origin; EA II: European Americans of Hispanic Origin; Num-Sample: the number of subjects within each population

### Discussion

M^3^-*S* was motivated from a data set with HapMap samples genotyped as part of the study, but many studies do not have these valuable samples collected. In this case, we may use pairs of raw intensity of HapMap subjects provided by the International HapMap Project [3, 4], but raw data in the Affymetrix data format cannot be directly applied to our program. Although some researchers have successively transformed the Affymetrix raw data into the Illumina raw data with high consistency [19–21], we are not sure the impact of transformation on the final genotyping result.

M^3^-*S* applies the reference SNP selection step to samples with known genotypes for improving the missing rate and call accuracy for rare SNPs, especially for SNPs with very low MAF. The successful application of M^3^-*S* is to select the appropriate Ref featured SNP from the whole genome. In practice, it is not practical to search the Ref featured SNPs from the entire genome due to plenty of tedious calculation involved, then the instrumental featured SNPs near the target featured SNP are picked out. The assumption about identical probe response of all SNPs allows each SNP to borrow information from other good quality SNPs. When some probes break this assumption, searching for the most optimal Ref featured SNP is still an open question. Besides, the reference SNP selection is based on maximizing the mathematical similarity between the target featured SNP and the Ref featured SNP. Because the probe intensity is highly correlated with 50 base probe sequence, incorporating the probe sequence information in the reference SNP selection procedure may greatly improve the quality of SNP calls.

The prerequisite for running M^3^-*S* is based on the collection of samples with known genotypes. However, some SNP-array data may not contain individuals with known genotypes, which results in the restricted use in M^3^-*S*. Fortunately, M^3^-*S* is a supplement to our previous method M^3^ [17]. We strongly suggest scientists to use M^3^ method if their SNP array data do not contain any HapMap samples. If the data have samples with known genotypes, they could apply M^3^-*S*. Scientists could try these methods according to their requirements. Recently, a large amount of rare variants are widely captured in many SNP array data, some new powerful calling algorithms have been proposed for accurately calling rare SNPs, such as: optiCall [22] and zCall [11]. To better understand the effectiveness of various calling algorithms, we consider to summarize and compare the performances of multiple popular calling methods in our future study for providing an application guide.

## Conclusion

Most genotyping approaches for microarray data are population-based methods, e.g. GenCall with GenTrain, that infer genotype clusters from a large number of samples to achieve a certain call accuracy. Although it may work well for common SNPs, it may perform poorly for rare SNPs where genotype clusters cannot be reliably established unless a very large number of samples are available. A SNP-based method, GenoSNP, was designed to address this problem, but many more SNPs inferred from GenoSNP tend to fail the HWE test which indicates the violation of the strong underlying assumption for GenoSNP to succeed. Recently, we proposed M^3^ to combine the benefits of both population-based and SNP-based strategies. Although M^3^ outperformed other methods, it is not able to use samples with known genotype information when they are available in a study, often as quality control samples. In this study, we propose M^3^-*S* to take advantage of genotype cluster information from the samples with known genotypes to further improve M^3^ on genotyping at rare SNPs. *M* ^3^-*S* is a two-stage procedure where the reference SNP selection method is applied to samples with known genotypes at rare SNPs to estimate the parameters of the three genotype clusters, followed by simulating additional samples from the Gaussian mixture distribution, and fitting GMM to the augmented data for genotype calling. The superiority of this method rests on two aspects. First, samples with known genotypes help define the boundaries of three genotype clusters before finally genotyping. Second, adding simulated data in the original population greatly enlarges the sample size while genotyping. These two aspects help us improve genotyping technique for rare SNPs.

## Additional file

Additional file 1
**Supplemental Materials.** A: Concordance among replicates of HapMap samples. B: Computational time of M^3^-*S*. C: Comparison of different measures in reference SNP selection, D: Comparison of different calling methods. E: The effect of the threshold. (PDF 89 kb)
