# Supplementary material for: M3-S: a genotype calling method incorporating information from samples with known genotypes
Source: BMC Bioinformatics. 2015 Dec 3;16:403. doi: 10.1186/s12859-015-0824-5 (PMC4669649; doi:10.1186/s12859-015-0824-5)
Supplement: Additional file 1 — Supplemental Materials. A: Concordance among replicates of HapMap samples. B: Computational time of M3-S. C: Comparison of different measures in reference SNP selection, D: Comparison of different calling methods. E: The effect of the threshold. (PDF 89 kb) [file 12859_2015_824_MOESM1_ESM.pdf]

Supplemental Materials

A: Concordance among replicates of HapMap samples

Table 1: Comparison of discordance counts among GenCall, GenoSNP, M^3 and M^3-S

Count	GenCall	GenoSNP	M^3	$M^3_{85\%} - 3000$
# of discrepancy	2379	9465	7228	2954
# of missing SNPs	366	0	0	0
# of missing observations	2207	12253	3368	4489

Note that # of discrepancy: the total number of discordance among repeated subjects; # of missing SNPs: the entire SNPs are missing; # of missing observations: partial observations are missing within SNPs; $M^3_{85\%} - 3000$: M^3 incorporating samples with known genotypes plus 3000 simulated subjects under 85% threshold.

Overall, there are 38 different HapMap samples, and the number of replications for each HapMap sample varies from 1 to 33. The number of discordance among repeated subjects are recorded for various methods. If a missing observation among repeated individuals is observed, the number of discrepancy is only calculated from non-missing observations. In general, GenCall has the largest number of missing observations including 366 entire missing SNPs and 2207 missing observations (The total missing observations are 53813), followed by GenoSNP, M^3-S and M^3. It is clearly seen that GenCall gives the smallest number of discordance, but provides the largest number of missing observations. M^3-S gives the second smallest number of discordance among replications, and a much smaller number of missing observations, compared to M^3 and GenoSNP.

B: Computational time of M^3-S

Note that the average speed of M^3-S with 600 simulated subjects is around 0.0409 seconds per SNP, and the average speed of M^3-S with 3000 simulated individuals is 0.0436 seconds per SNP. It is clearly seen that the computational time increases when we enlarge the number of simulated samples. In practice, we strongly recommend scientists to split whole genome intensity data into chromosomes and genotype chromosome-level intensity data separately in parallel on different CPUs to significantly save computational time.

Table 2: Summary of computational time of M^3-S method

Workstation	Sample Size	# SNP	Total Time (second)	Time per SNP (second)
RAM: 32GB	3258+600	250	10.3066	0.0412
System: 64 bit		500	19.9270	0.0399
CPU: 2.40GHz		10000	386.033	0.0386
		20000	877.0302	0.0439
	3258+3000	250	10.8441	0.0434
		500	21.6353	0.0433
		10000	415.8256	0.0416
		20000	918.1505	0.0459

Note: 3258+600: 3258 original study population plus 600 simulated subjects; 3258+3000: 3258 original study population plus 3000 simulated subjects; 10000 SNPs are from chromosome 22; 20000 SNPs are from chromosome 20.

C: Comparison of different measures in reference SNP selection

Table 3: Comparison of computational time for different measures

Workstation	Sample Size	Measure	Average time per SNP (second)
RAM: 32GB	3258+3000	Maholanobis	0.0436
System: 64 bit, CPU: 2.40GHz		Cluster	0.0714

Note: 3258+3000: 3258 original study population plus 3000 simulated subjects; Maholanobis: Maholanobis distance; Cluster: cluster distance defined in M^3 method.

In practice, we tried to apply the cluster measure in this manuscript, and found that the improvement is not remarkable. But the computational time of the cluster is longer than that of the maholanobis distance (Supplemental Table 3). We have to balance between the selection of measures and the computational speed.

D: Comparison of different calling methods

Despite the overall high concordance rates (Table 4 in the main paper), there are some discrepancies among these three algorithms. In Supplemental Table 4, the concordance broken down to specific genotypes, i.e. major homozygote, heterozygote, and minor homozygote, is summarized when the null genotypes are excluded from the comparisons. We note that the

major homozygote calls by M^3-S is more frequently called heterozygote by GenoSNP. For example, 0.22% of genotypes called as major homozygote by M^3-S are called heterozygote by GenoSNP, but only 0.03% of genotypes as major homozygote by GenoSNP are called heterozygote by M^3-S. Figure 3 (d- f) [1] clearly shows why GenoSNP likely calls homozygote genotype in heterozygote.

Table 4: The concordance and discordance rates of both homozygotes and heterozygotes among GenCall, GenoSNP, M^3 and $M^3_{85\%} - 3000$

		$M^3_{85\%}$-3000 (%)		
Algorithm		Major-Homo	Heter	Minor-Homo
GenCall (%)	Major-Homo	63.23	0.02	≈ 0
	Heter	$\approx$0	28.98	0.02
	Minor-Homo	≈ 0	0.01	7.65
GenoSNP (%)	Major-Homo	62.94	0.03	0.09
	Heter	0.22	29.03	0.08
	Minor-Homo	0.04	0.08	7.48
M^3 (%)	Major-Homo	63.08	0.13	0.04
	Heter	0.11	28.90	0.04
	Minor-Homo	0.02	0.01	7.59

Note: $M^3_{85\%}$-3000: M^3 incorporating samples with known genotypes plus 3000 simulated samples under 85% threshold; Major-Homo: major homozygote; Heter: heterzygote; Minor-Homo: minor homozygote.

We use two examples to summarize the different performances of various calling methods. For rs1000427, M^3-S calls 4 dark red observations in heterozygote group, but GenCall calls these 4 subjects in missing group. For rs1009730, M^3-S calls 1 dark red subject in heterozygote group, but GenoSNP calls this individual in missing group; M^3-S calls 2 green subjects in major homozygote group and missing group, but GenoSNP calls these 2 subjects in heterzygote group.

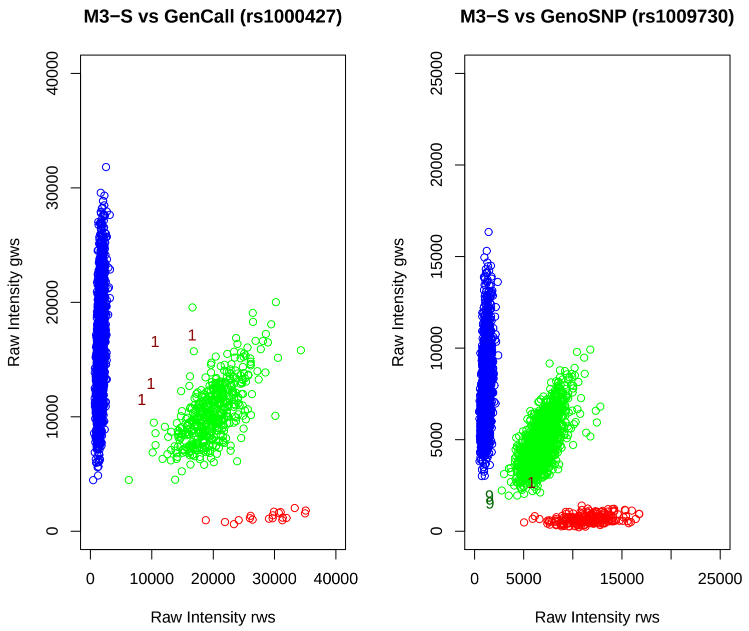

Figure 1: Calling differences among GenCall, GenoSNP and M^3-S.

E: The effect of the threshold

One review literature suggested that the GenoSNP threshold should be at least 80% to achieve good quality calling result [2]. It also shows that extremely high quality of genotyping is based on 95% threshold, but may lead to many missing observations in SNP calling, especially for rare SNPs. When the cutoff is reduced to 70%, more false positive calling results may be collected. Therefore, after exploring different thresholds, 85% was selected in our comparisons. To compare these methods using different thresholds, we summarize the results with thresholds 70% and 85% in Supplemental Table 5. Overall, the results are quite robust to different thresholds.

Table 5: Comparison of call rates and concordance under different thresholds

Design	Sample Size	Item	$M^3_{70\%}$ %	$M^3_{85\%}$ %
2:1	3258+3000	Call Rate	99.73	99.65
		Accuracy	99.40	99.38

Note: 2:1: 94 individuals are in the training set, and 47 subjects are in the testing group; 3258+3000: 3258 original study population plus 3000 simulated subjects; $M^3_{70\%}$: M^3 incorporating samples with known genotypes under 70% threshold; $M^3_{85\%}$: M^3 incorporating samples with known genotypes under 85% threshold; Call Rate: the percentage of valid genotypes; Accuracy: the percentage of consistent genotype;

References

[1] Giannoulatou,E.et al. GenoSNP: a variational Bayes within-sample SNP genotyping algorithm that does not require a reference population. Bioinformatics. 2008; 24,2209-2214.

[2] Ritchie,M.E. et al. Comparing genotyping algorithms for Illumina's Infinium whole-genome SNP BeadChips. BMC Bioinformatics. 2011, 12: 68.
